# Depression, anxiety, and performance status among the women with metastatic breast cancer receiving palliative care in Bangladesh: A cross sectional study

**DOI:** 10.1002/hsr2.911

**Published:** 2022-10-29

**Authors:** Nashid Islam, Jheelam Biswas, Mostofa Monwar Kowshik, Md Maruf Ahmed Molla, Mridul Saker, Mostofa Kamal Chowdhury, AKM Motiur Rahman Bhuiyan, Nezamuddin Ahmad

**Affiliations:** ^1^ Department of Palliative Medicine Bangabandhu Sheikh Mujib Medical University (BSMMU) Dhaka Bangladesh; ^2^ Department of Medicine Dhaka Medical College Dhaka Bangladesh; ^3^ Department of Microbiology and Immunology SUNY Upstate Medical University New York United States

**Keywords:** anxiety, Bangladesh, breast cancer, depression, ECOG status, palliative care

## Abstract

**Background and Aims:**

Advanced breast cancer patients suffer from various psychological issues including depression and anxiety. This study aims to explore these psychological issues and their relationship with the performance status.

**Methods:**

This cross‐sectional study was conducted among 95 patients with metastatic breast cancer attending the Department of Palliative Medicine, Bangabandhu Sheikh Mujib Medical University, Bangladesh from April 2021 to September 2021. Data was collected by face‐to‐face interview using a structured questionnaire along with Hospital Depression and Anxiety Scale. The performance status of the patients was determined by the Eastern Cooperative Oncology Group (ECOG) performance scale. The association between different variables were assessed by *χ* ^2^ test and Fisher Exact test.

**Result:**

Mean age of the respondents was 48.9 ± 9.9 years. Most of them were married (94.7%), muslim (92.6%) and homemakers (82.1%). More than half (52.6%) of the patients were evaluated having ECOG performance status grade II. Four out of ten  (44.2%) patients had moderate to severely anxiety, and almost one‐third (36.9%) patients were suffering from moderate to severe depression. The patients with high educational status were found to have less depression. In addition, patients faring better on ECOG performance scale (Grade 0 to I) had significantly (*p* < 0.05) less depression and anxiety.

**Conclusion:**

Depression and anxiety are one of the major psychological sufferings among the women with metastatic breast cancers. All women suffering from breast cancer should be routinely screened and assessed for phychological distress and ensure early intervention.

## 1 BACKGROUND

Breast cancer is the highest occurring cancer worldwide and has surpassed lung cancer with an estimated 2.3 million new cases in 2020, which represents 11.7% among cancer cases. It is the fifth leading cause of cancer death globally, with 685,000 deaths. Breast cancer is the most common form of cancer among women, accounting for one in four new cases and one in six cancer‐related deaths globally (159 of 185 countries).^1^ Almost 6% of these patients have metastasis during the first diagnosis.^2^ It has also been reported to be the highest prevalent (about 19.3 per 100,000) malignancy among Bangladeshi women between 15 and 44 years of age.^3^

The rising incidence rates reflect a higher prevalence of reproductive and hormonal risk factors (early age at menarche, late age at menopause, first birth at an advanced age, taking less number of children, less or not breastfeeding, hormone therapy at menopause, taking oral contraceptives) and lifestyle risk factors (increase alcohol intake, excessive body weight, sedentary lifestyle), as well as increased awareness and detection through screening. The incidence rates of newly diagnosed breast cancer are 88% higher in developed countries than in the developing countries (55.9 and 29.7 per 100,000, respectively).^4^

Currently, breast cancer is prevented through screening (mammography and MRI), chemoprevention using selective estrogen receptor modulators and the aromatase inhibitors and biological prevention (using Herceptin and pertuzumab).^5^

The female breast is considered as an iconic representation of a woman's femininity in the society, the organ of beauty, sexuality, and motherhood. Diagnosis, treatment modalities and their side effects of breast cancer have tremendous psychological impacts on the patients.^6^ These symptoms include fear of death, emotional distress, body image disturbance, and disrupted social status.^7^

In Bangladesh, late presentation of breast cancer is very common. Along with the various physical symptoms these patients suffer from different psychological symptoms such as depression, anxiety, and melancholy.^8^ In addition, the effects of surgery and other disease modifying treatments lead to a significant psycho‐spiritual suffering.

Psychological sufferings vary in different stages of breast cancer. Patients experience various emotional and spiritual distresses related to appearance and changing roles in the family.^9^ This intense suffering caused by multiple unresolved symptoms lead towards a higher risk of having severe anxiety, depression, and possible mood disorders among these patients.^8^, ^10^ Almost 32.8% of the advanced breast cancer patients suffer from depression as well as 40% of them suffer from anxiety and sadness.^11^ A study among Bangladeshi breast cancer patients shows that, sometimes the diagnosis itself leads to divorce, rejection by the community, even acts as a motivation for suicide.^12^

Despite having increased psychological morbidity and significant depressive symptoms among the advanced breast cancer patients, these conditions are frequently underestimated or remain untreated in health care settings. This disparity lowers their quality of life, performance status, and survival rates.^13^ Because women with metastatic breast cancer live longer, the psychological caring for these patients has become a serious concern for both the oncologists and the palliative care teams. Studies demonstrate that initiation of early palliative care, integrated with oncology care, not only improves the psychological sufferings but also enhances coping among the patients with advanced cancer.^14^ According to the patient register (manually checked), almost 28% of the patients receiving palliative care in Department of Palliative Medicine of Bangabandhu Sheikh Mujib Medical University, were diagnosed with advanced breast malignancy. But little is known regarding the psychological sufferings of these patients. This study aims to explore anxiety and depression among these patients and determine their relationship with the patients' performance status.

## 2 METHODS

### 2.1 Study design and setting

This cross‐sectional study was conducted among the female patients diagnosed with metastatic breast cancers attending the in‐patient and outpatient service of the Department of Palliative Medicine, Bangabandhu Sheikh Mujib Medical University (BSMMU), Shahbag, Dhaka. Data collection was done from April 2021 to September 2021.

### 2.2 Sampling criteria

Patients having metastatic (Stage IV) breast cancer above 18 years of age getting palliative care were included in the study. The patients with pre‐existing disorders such as stroke, motor neuron disease, and so forth, which clearly interfere with the cognition, were excluded from this study.

### 2.3 Sample size

In a previous study the acceptance level of the disease condition was found to be 12.89 ± 2.37.^15^ With a 6% change in the acceptance level our calculated sample size was 74. We initially selected 103 patients from the hospital register by the simple random sampling‐27 from the in‐patient service, and 76 patients from the outpatient. During the data collection process, six of them developed some cognitive impairment, and two of them withdrew from the study due to physical condition. So, our final sample size was 95.

### 2.4 Data collection procedure

Data was collected by the principal investigator using a structured questionnaire containing sociodemographic variables, disease status, treatment‐related variables and Eastern Cooperative Oncology Group (ECOG) performance scale. After protocol and questionnaire development as well as obtaining the ethical approval, pretesting of the questionnaire was done among 10 breast cancer patients (10% of the total sample size). Necessary corrections were made based on the response and the instrument was finalized. The validated Bangla version of the “Hospital Depression and Anxiety Scale” was used to obtain the anxiety and depression of the patients.

ECOG Performance Status Scale was developed by the ECOG, now the ECOG‐ACRIN Cancer Research Group, and published in 1982.^16^ The validity and reliability of ECOG scale were evident when evaluated among physicians. They can, therefore, be used as a criterion in clinical trials for patients with cancer because they clearly define the physical status and medical requirements. There was a good correlation between physicians and patients using ECOG scale. Indeed, coefficients greater than 0.6 indicated a good validity of self‐evaluation related to the external criterion of the physician.^17^

The performance status is divided into five grades ranging from “0” to “V.” Grade 0 refers to the patients who are completely asymptomatic, fully active, and are able to carry out all predisease activities without restriction. Grade I refers to the patients who are symptomatic but completely ambulatory, with restrictions regarding physically strenuous activity, but are able to carry out work of a light or sedentary nature (e.g., lighthousework, officework). Grade II refers to the patients who are symptomatic, spends <50% in bed during the day, are ambulatory and capable of all self‐care but unable to carry out any work activities; and remain awake more than 50% of waking hours. Grade III refers to the patients who are symptomatic, spend >50% in bed, but not bedbound, are capable of only limited self‐care and confined to bed or chair 50% or more of waking hours. Grade IV refers to the patients who are completely bedbound and disabled. Grade V refers to death.^16^

The third part contains the validated “Bangla version of the Hospital Depression and Anxiety Scale (HADS) questionnaire.” The HADS contains seven items that assess anxiety and seven items that assess depression. HADS‐A or HADS‐D score of 8 was defined as a case, score from 8 to 10 is mild, from 11 to 14 is of moderate intensity, and above 14 denotes severe.^18^

Data was collected through face‐to‐face interviews. The principal investigator sat with the patients whenever they visit our inpatient or outpatient department, and with due permission asked the questions. The most appropriate response was noted in the questionnaire by the researcher. When the patient was unable to follow a question or response verbally, the caregiver was requested to give proper information. The duration of each interview was 30 minutes to 1 hour. Two to three patients were interviewed each day. Very frail patients were given multiple visits to complete an interview.

### 2.5 Data analysis

All statistical analyses were performed using the SPSS version 26. Descriptive analysis was done for the categorical variables like age, monthly family income, educational status, ECOG performance status, treatment history, and anxiety and depression scores.

Association between sociodemographic characteristics of the patients and depression and anxiety were assessed by *χ* ^2^ test and Fisher Exact test. Association between ECOG performance status and depression and anxiety were assessed by *χ* ^2^ and Fisher Exact test.

### 2.6 Ethical considerations

The ethical approval (Approval no: BSMMU/2021/3447, date: 17/04/2021) was obtained from the Institutional Review Board (IRB) of Bangabandhu Sheikh Mujib Medical University (BSMMU). Written informed consent was taken from all the eligible patients. Sensitive questions were discussed privately. As they were terminally ill patients, their health conditions were considered during data collection.

## 3 RESULTS

Mean age of the patients enrolled in the study is 48.9 ± 9.9 years. Most of them are married (94.7%), Muslim (92.6%), homemakers (82.1%) by profession, and had education levels above secondary (63.2%). Nearly half of them (68.4%) had no family history of malignancy. Most of them received disease‐modifying treatment like surgery (87.4%), radiotherapy (70.5%), and chemotherapy (96.8%) and 54.7% of patients received alternate therapy (Table 1).

**Table 1:** **Sociodemographic characteristics of the patients (*n*** = **95)**

Sociodemographic characteristics	Frequency (*n*)	Percentage (%)	
Age (in years)			
Up to 40	21	22.1	
41–50	37	38.9	
51–60	24	25.3	
>60	13	13.7	
Mean (years)	48.9 Years		
Educational status			
No formal education	15		15.8
Up to primary	12		12.6
Secondary	8		8.4
Above secondary	60		63.2
Occupational status			
Homemakers	78		82.1
Service holder	17		17.9
Income group (in BDT)^a^			
Lower middle (8000–30,0000)	28		29.5
Upper middle (31,000–92,000)	24		25.3
High (>92,000)	21		22.1
Marital status			
Married	90		94.7
Widow/separated/unmarried	5		5.3
Religion			
Islam	88		92.6
Hinduism and Christianity	7		7.4
Family history of malignancy			
Yes	30		31.6
No	65		68.4
Mode of treatment along with palliative care			
Chemotherapy	92		96.8
Radiotherapy	67		70.5
Surgery	83		87.4
Hormone therapy	15		15.8
Alternate therapy	52		54.7

^a^ According to World Bank, 2021.

According to ECOG performance status, more than half (52.6%) of the patients were symptomatic but completely ambulant (Grade II). A few (6.3%) were completely bedbound (Grade IV) (Table 2).

**Table 2:** Eastern Cooperative Oncology Group (ECOG) performance status of the patients (*n* = 95)

ECOG performance status	Frequency (*n*)	Percentage (%)
Grade 0	15	15.8
Grade 1	50	52.6
Grade 2	15	15.8
Grade 3	9	9.5
Grade 44	6	6.3

Nearly half of the patients (47.4%) patients had no anxiety according to the HADS scale, while 4 out of 10 (44.2%) patients were considered as moderate to severely anxious. Again, almost half (51.6%) of the patients had no depression while 1 out of 10 (11.5%) patients are suffering from mild, and 3 out of 10 (36.9%) were suffering from moderate to severe depression (Table 3).

**Table 3:** Anxiety and depression among the patients (*n* = 95)

	Frequency (*n*)	Percentage (%)
Anxiety		
No	45	47.4
Mild	8	8.4
Moderate	27	28.4
Severe	15	15.8
Depression		
No	49	51.6
Mild	11	11.5
Moderate	15	15.8
Severe	20	21.1

It had been found that that patients with high educational status had less depression compared to others (*p* = 0.048). We didn't find any significant association between anxiety and sociodemographic characteristics (Table 4).

**Table 4:** Association between sociodemographic characteristics of the patients and depression (*n* = 95)

Variables	Psychological symptoms		*p* Value
	Absent	Present	
	Depression		
Age (in years)			
Up to 40	14 (23.3%)	7 (20.0%)	0.111^a^
41–50	18 (30.0%)	19 (54.3%)	
51–60	19 (31.7%)	5 (14.3%)	
>60	9 (15.0%)	4 (11.4%)	
Educational status			
No formal education	6 (10.0%)	9 (25.7%)	**0.048** a
Primary (I–V)	6 (10.0%)	6 (17.1%)	
Secondary (6–12)	4 (6.7%)	4 (11.4%)	
Higher Secondary	44 (73.3%)	16 (45.7%)	
Occupational status			
Homemakers	46 (76.7%)	32 (91.4%)	0.097
Service holder	14 (23.3%)	3 (8.6%)	
Income group (in Tk)			
Lower middle (8000– 30,0000)	45 (75.0%)	30 (85.7%)	0.337^a^
Upper middle (31,000–92,000)	11 (18.3%)	5 (14.3%)	
High (>92,000)	4 (6.7%)	0 (0.0%)	
	Anxiety		
Age (in years)			
Up to 40	8 (17.8%)	13 (26.0%)	0.054
41–50	13 (28.9%)	24 (48.0%)	
51–60	15 (33.3%)	9 (18.0%)	
>60	9 (20.0%)	4 (8.0%)	
Educational status			
No formal education	4 (8.9%)	11 (22.0%)	0.217^a^
Up to primary	5 (11.1%)	7 (14.0%)	
Below secondary	3 (6.7%)	5 (10.0%)	
Secondary passed and above	33 (73.3%)	27 (54.0%)	
Occupational status			
Homemakers	36 (80.0%)	42 (84.0%)	0.789
Service holder	9 (20.0%)	8 (16.0%)	
Income group (in Tk)			
Lower middle (8000– 30,0000)	33 (73.3%)	42 (84.0%)	0.103^a^
Upper middle (31,000–92,000)	8 (17.8%)	8 (16.0%)	
High (>92,000)	4 (8.9%)	0 (0.0%)	

*Note*: *χ* ^2^ test was done. Bold value indicates significant *p* < 0.05.

^a^ Fisher Exact test was done.

Almost 13.3% patients with ECOG performance status grade 0% and 66.4% with ECOG status grade I did not have depression. It was also evident from the result that the patients with less symptoms are less depressive compared to others (*p* = 0.002). More than half (68.9%) of the patients having ECOG performance status grade I did not have anxiety. It had been also found that that patients with less symptoms were less anxious compared to others (*p* = 0.001) (Table 5).

**Table 5:** Association between Eastern Cooperative Oncology Group (ECOG) performance status and depression (*n* = 95)

ECOG performance status	Psychological symptoms		*p* Value
	Absent	Present	
	Depression		
Grade O	8 (13.3%)	7 (20.0%)	
Grade I	40 (66.4%)	10 (28.6%)	
Grade II	7 (11.7%)	8 (22.9%)	**0.002** a
Grade III	2 (3.3%)	7 (20.0%)	
Grade IV	3 (5.0%)	3 (8.6%)	
	Anxiety		
Grade O	7 (15.6%)	8 (16.0%)	**0.001** a
Grade I	31 (68.9%)	19 (38.0%)	
Grade II	5 (11.1%)	10 (20.0%)	
Grade III	1 (2.2%)	8 (16.0%)	
Grade IV	1 (2.2%)	5 (10.0%)	

*Note*: Bold values indicates significant *p* < 0.05.

^a^ Fisher Exact test was done.

## 4 DISCUSSION

Women with breast cancer often face various psychological issues. Studies show that, Stage IV breast cancer patients suffer from multitude of mental health conditions.^19^ Among them depression and anxiety are most prevalent.^20^ In this study, nearly half (44.2%) of the patients had moderate to severe anxiety, and almost one‐third (36.9%) had moderate to severe depression. All the patients got available palliative care including physical symptom management and psychospiritual support from the attending palliative care team. This finding is on par with the previous studies conducted among the Bangladeshi women with incurable diseases.^21^ Most of the participants in our study belonged to middle age (40–60 years) group, practice Islamic religious faith, and are homemakers by profession. They are not usually economically self‐dependent, burdened with social taboos, as well as different cultural and religious stigmas. Still, the prevalence of anxiety and depression is significantly lower among these women, compared to the countries with the same religious and social structure such as Pakistan, where almost 65% of the advanced breast cancer patients suffer from varying level of anxiety and depression.^22^ A study done by Rahman et al.^23^ found that Bangladeshi women experience significant emotional turmoil as well as cognitive issues (such as memory and concentration issues) after breast surgery.

One significant finding was that women with higher educational status had significantly less anxiety and depression. It supports the finding of an Iranian study where women having less or no education had significantly more chances of developing anxiety and depression.^13^ It is possible that, patients with higher educational status have more opportunities to be aware of their disease condition and find different coping mechanisms to deal with their situation.

Here, another interesting finding was with the ECOG performance status and psychological symptoms. Women with better performance status had a low level of depression and anxiety, while other studies also found that the patients with poor performance status are more prone to developing anxiety and depression.^22^, ^24^ Though we have not done in‐depth exploration of this situation, but it has been observed that patients with poor physical condition feel more emotionally vulnerable than those who have comparatively better physical condition. Also, being able to perform daily tasks such as self‐care, give the patients more confidence and freedom to deal with their disease.

Although no significant relationship between economic and marital status with psychological issues was found, different studies suggest that unmarried women have less depression than married women. It is also suggested that being unmarried act as a protective factor for developing psychological trauma as they have less worry regarding family responsibility.^13^ On the contrary, another study in Turkey showed that, strong family tie acts as a strong factor in decreasing breast cancer‐related morbidity.^25^ Still, we can't draw any conclusion, as majority (95%) participants in our study were married. So, there is a lack of sufficient data to establish any significant relationship between marital status and psychological issues of our patients. Also, no significant relationship had been found with economic condition and psychological issues in our study. But it was observed that financial uncertainty often had negative impacts on the mental health of the cancer patients.^26^

Despite the increasing burden of mental health issues among the cancer patients, the interdisciplinary approach between mental health, oncology and palliative care is uncommon in Bangladesh, and the psychosocial and mental health problems of breast cancer patients are frequently overlooked. Only a few scattered private hospitals, mental health clinics, and non‐profit organizations are trying to provide mental health care to cancer patients. Existing organized psychosocial services in large tertiary hospitals and oncology settings are far away from meeting the demands.^27^ However, in our department, the attending physicians, nurses and palliative care assistants try to address some of these psychological issues of the advanced breast cancer patients referred from various oncology centers. We acknowledged and listened to their problems by letting them vent their feelings. Still, we don't have any trained professional psychologist, psychiatrist, or chaplain on our team. Thus the care provided to these patients may not address the whole extent of their mental and spiritual sufferings.

This study has several limitations. The major drawback of this study is that the sample was selected from only one palliative care center, so the results of this study cannot be generalized. In addition to that, some confounding factors had not been controlled. For instance, past psychiatric history or other psychological issues, which might precipitate anxiety and depression among these patients were not explored. Also, some of the respondents did not give their exact feedback due to the fear of negative consequences, thus skewing the results. Despite having the above limitations, this study gives us an overall picture of two major psychological issues among the advanced breast cancer patients receiving palliative care in our country.

## 5 CONCLUSION

Depression and anxiety are one of the major psychological sufferings among the women with metastatic breast cancers. All women suffering from breast cancer should be routinely screened and assessed for phychological distress. Recognition of anxiety and depression, and early intervention by mental health professionals familiar with the care of cancer patients may help improving quality of life.

## AUTHOR CONTRIBUTIONS

**Nashid Islam**: Conceptualization; data curation; formal analysis; funding acquisition; investigation; methodology; project administration; resources; software; validation; visualization; writing–original draft; writing–review & editing. **Jheelam Biswas**: Data curation; formal analysis; methodology; validation; writing–original draft; writing–review & editing. **Mostofa Monwar Kowshik**: Data curation; investigation; project administration; supervision; writing–original draft; writing–review & editing. **Maruf Ahmed Molla**: Formal analysis; methodology; supervision; visualization; writing–original draft; writing–review & editing. **Mridul Saker**: Data curation; formal analysis; funding acquisition; investigation; methodology; project administration; resources; software; writing–original draft. **Mostofa Kamal Chowdhury**: Conceptualization; formal analysis; investigation; methodology; project administration; resources; software; supervision; visualization; writing–original draft; writing–review & editing. **AKM Motiur Rahman Bhuiyan**: Conceptualization; data curation; investigation; methodology; project administration; resources; software; supervision; visualization; writing–original draft; writing–review & editing. **Nezamuddin Ahmad**: Conceptualization; investigation; methodology; project administration; resources; software; supervision; validation; visualization; writing–original draft; writing–review & editing. All authors have read and approved the final version of the manuscript.

## CONFLICT OF INTEREST

Dr. Md. Maruf Ahmed Molla is an Editorial Board member of Health Science Reports, and a coauthor of this article. To minimize bias, he was excluded from all editorial decision‐making related to the acceptance of this article for publication. The remaining authors declare no conflict of interest.

## TRANSPARENCY STATEMENT

The lead author Dr. Nashid Islam affirms that this manuscript is an honest, accurate, and transparent account of the study being reported; that no important aspects of the study have been omitted; and that any discrepancies from the study as planned (and, if relevant, registered) have been explained.

## Data Availability

All data relevant to the study are accessible in Mendely data 10.17632/95h2d6pdnj.1. The lead author Dr. Nashid Islam has full access to all of the data in this study and takes complete responsibility for the integrity of the data and the accuracy of the data analysis.
